# Myelofibrosis as an Unusual Cause of Back Pain: A Case Report

**DOI:** 10.7759/cureus.83775

**Published:** 2025-05-09

**Authors:** Richard Bechert

**Affiliations:** 1 Integrative and Complementary Medicine, Victory Health, Guntersville, USA

**Keywords:** back pain, case report, flank pain, integrative care, myelofibrosis

## Abstract

Low back pain and, to a lesser degree, associated flank pain are common presentations to an integrative musculoskeletal clinic. Most cases have a neuromusculoskeletal or, less commonly, a urological etiology. Myelofibrosis is a chronic, uncommon blood neoplasm. Here, we describe the clinical presentation and diagnostic workup of a 76-year-old male who complained of back and side pain that did not respond to an initial trial of care. The case is followed through the diagnostic process, leading to the final diagnosis of myelofibrosis. This case emphasizes the need for further workup and a broad-spectrum differential diagnosis in cases of back and flank pain that do not respond to care, including consideration of myelofibrosis as a rare etiology.

## Introduction

Low back pain is among the most common complaints, affecting 54-90% of the population [1]. It is the second most common reason for seeking primary care [2]. Low back pain can originate from inflammatory, mechanical, urological, abdominal, neoplastic, metabolic, degenerative, non-specific, or vascular etiologies [3].

There are warning signs in a patient’s history and physical examination that warrant diagnostic laboratory testing, imaging, or both. Indicators for further workup include age over 50 years, a history of malignancy, trauma, risk for osteoporotic compression fracture, nocturnal pain, unexplained weight loss, night sweats, fever, recent infection, intravenous drug use, unrelenting pain, incontinence, high risk of developmental disorders, high risk for acute spondylolisthesis, chronic steroid use, saddle anesthesia, bilateral neurological symptoms, cauda equina compression, motor weakness, diminished reflexes, weak anal sphincter, and tenderness over the spinous processes in patients at risk for fracture [4].

Most cases of back pain are mechanical, degenerative, or biopsychosocial in nature. Common presentations of myelofibrosis include fatigue, night sweats, abdominal pain, weakness, and anemia [5]. This case report describes a 76-year-old man who presented to an integrative medicine clinic with low back and flank pain and was ultimately diagnosed with myelofibrosis. The report highlights the symptoms and clinical reasoning that led to an accurate diagnosis and discusses potential missteps in identifying this rare condition, which can mimic more common musculoskeletal disorders.

## Case presentation

A 76-year-old male reported lumbar and bilateral sacroiliac pain rated at 6/10, which increased to 8/10 with movement. The onset of pain was gradual for one week and was described as aching and sharp. He had a prior history of lumbar back pain. His current pain decreased while standing. He had taken acetaminophen and hydrocodone, prescribed by his primary care provider, and had undergone no recent diagnostic tests. Social history was negative for tobacco, alcohol, or illicit drug use. Family history was positive: his mother had Parkinson’s disease, and both his father and brother had a history of cancer. He reported no recent abnormal weight changes. His medical history was positive for hypertension, congestive heart failure, and kidney failure. He had a spine functional index of 36. His blood pressure was 160/72, and he was oriented to time, place, and person.

Positive orthopedic findings included a positive flexion, abduction, and external rotation (FABER) test on the right; reduced right hip motion compared to the left; and a positive flexion, adduction, and internal rotation (FADIR) test on the left. FABER and FADIR are common tests for hip and sacroiliac joint dysfunction. His lower extremity reflexes were 2/2, and muscle strength was 5/5. His lumbar range of motion was limited to 5°-10° in all planes. He had trigger points in the right and left erector spinae muscles, and both lumbar and sacroiliac distraction techniques relieved his pain. His initial diagnosis was mechanical pain. He was treated with distraction manipulation and in-office electrical stimulation, and he was prescribed a home transcutaneous electrical nerve stimulation (TENS) unit. By the next day, his pain had decreased to 4/10. He underwent another distraction manipulation and was prescribed cat/cow stretching exercises. Despite this, he remained very uncomfortable and visited the nurse practitioner (NP) at the clinic, where he received an intramuscular injection of 6 mg dantrolene and was prescribed a Medrol Dosepak (4 mg) and methocarbamol (500 mg), both to be taken orally twice daily. Over the next two visits, his pain resolved, and he was released on a PRN (as-needed) basis.

**Figure 1:**
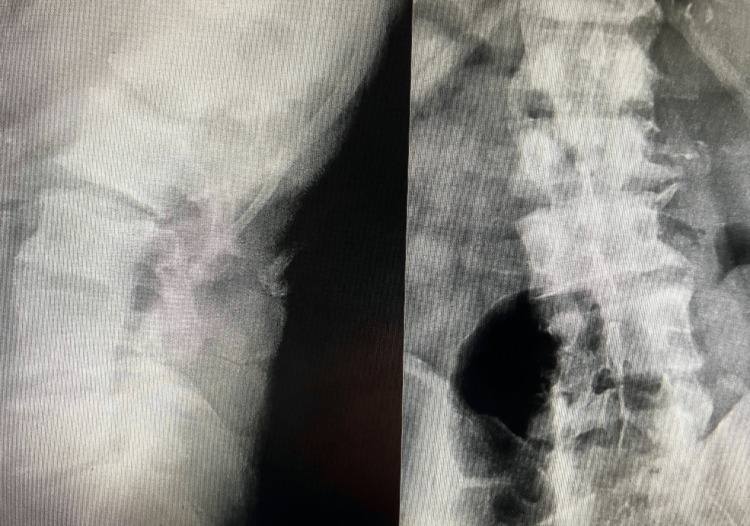
Lateral view of the lumbar spine on the left and anteroposterior view on the right demonstrating degenerative changes

Two weeks later, the patient returned with a complaint of 9/10 pain in the left lumbar region and left abdomen. He denied any trauma. His lumbar range of motion remained limited. His vital signs were as follows: glucose level of 154 mg/dL, temperature of 98.7°F, blood pressure of 126/61 mmHg, oxygen saturation of 92% on room air, and pulse of 92 bpm. He exhibited jump signs and guarding over the left flank and abdomen. His review of systems was negative. He had started taking 600 mg of ibuprofen four times daily, which provided minimal relief. He recalled a history of anemia, for which he had been treated by a hematologist six months earlier and subsequently released. A lumbar spine X-ray, lab workup, and CT scan of the pelvis and abdomen were ordered. The lumbar spine X-ray demonstrated degenerative changes in the lumbar spine (Figure 1).

**Figure 2:**
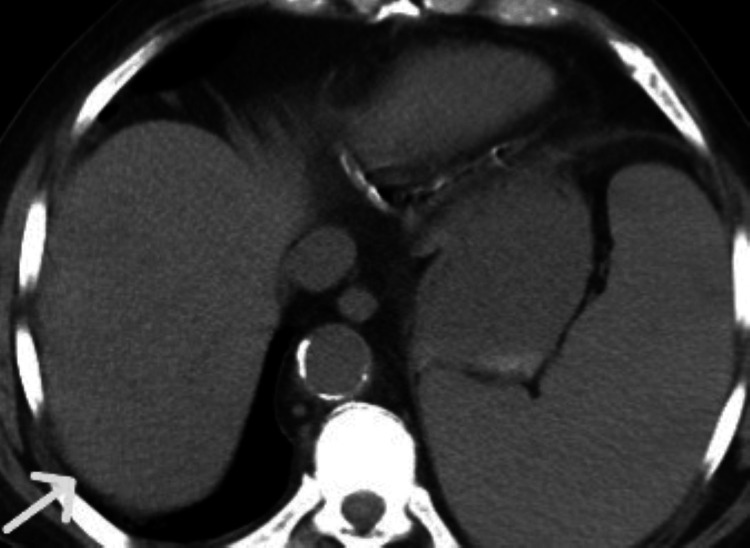
CT scan with enlarged spleen on the left CT: computed tomography

The CT revealed splenomegaly (Figure 2) and a small 3.8 cm abdominal aneurysm and acute diverticulitis.

His lab work was largely unremarkable, except for a slightly low glomerular filtration rate of 52 ml/min/1.73 m² (reference range: 60-90). A complete blood count revealed a mildly low red blood cell count of 3.64 × 10^6/μL (3.87-5.71), with hemoglobin at 9.9 g/dL (12.1-17.0). The immature granulocyte count was elevated at 1.75 × 10^3/μL (<0.06). The sedimentation rate was also elevated at 60 mm/hr (reference range: 0-20 mm/hr) (Table 1).

**Table 1:** Abnormal lab work

Lab test	Abnormal results	Normal range
Glomerular filtration rate ml/min/1.73 m2	52 ml/min/173 m2	60-90 ml/min/1.73 m2
Red blood count 10 x 6	3.64 10 x 6/u	3.87-5.71 10 x 6/u
Hemoglobin gm/dl	9.9 gm/dl	12.1-17.0 gm/dl
Immature granulocytes 10 x 3/u	1.75 10 x 3/u	0.00-0.06 10 x 3/u
Erythrocyte sedimentation rate mm/hr	60 mm/hr	20 mm/hr

The patient was injected by the NP with 6 mg of dexamethasone and prescribed 20 mg of prednisone for five days, along with methocarbamol. Following an oral report of a CT confirming diverticulitis, the patient was prescribed 500 mg of ciprofloxacin daily for seven days. Ibuprofen was discontinued due to the diverticulitis and the associated increased risk of bleeding. The working diagnosis was diverticulitis as the primary pain generator. After receiving the CT results, a consultation was initiated for diverticulitis, hernia, and anemia, and follow-up ultrasounds of the aorta and spleen were scheduled for six and three months, respectively.

Three weeks later, the patient returned to the chiropractor with increasing left flank pain. Vitals were benign, and no medication changes were made. Muscle strength was 5/5 in the lower extremities; however, the S1 reflex had decreased to 0/2, whereas it had previously been normal at 2/2. The patient had a limited extension range of motion and a trigger point in the left intercostal area. The working diagnosis at that time included possible intercostal pain in conjunction with diverticulitis.

**Figure 3:**
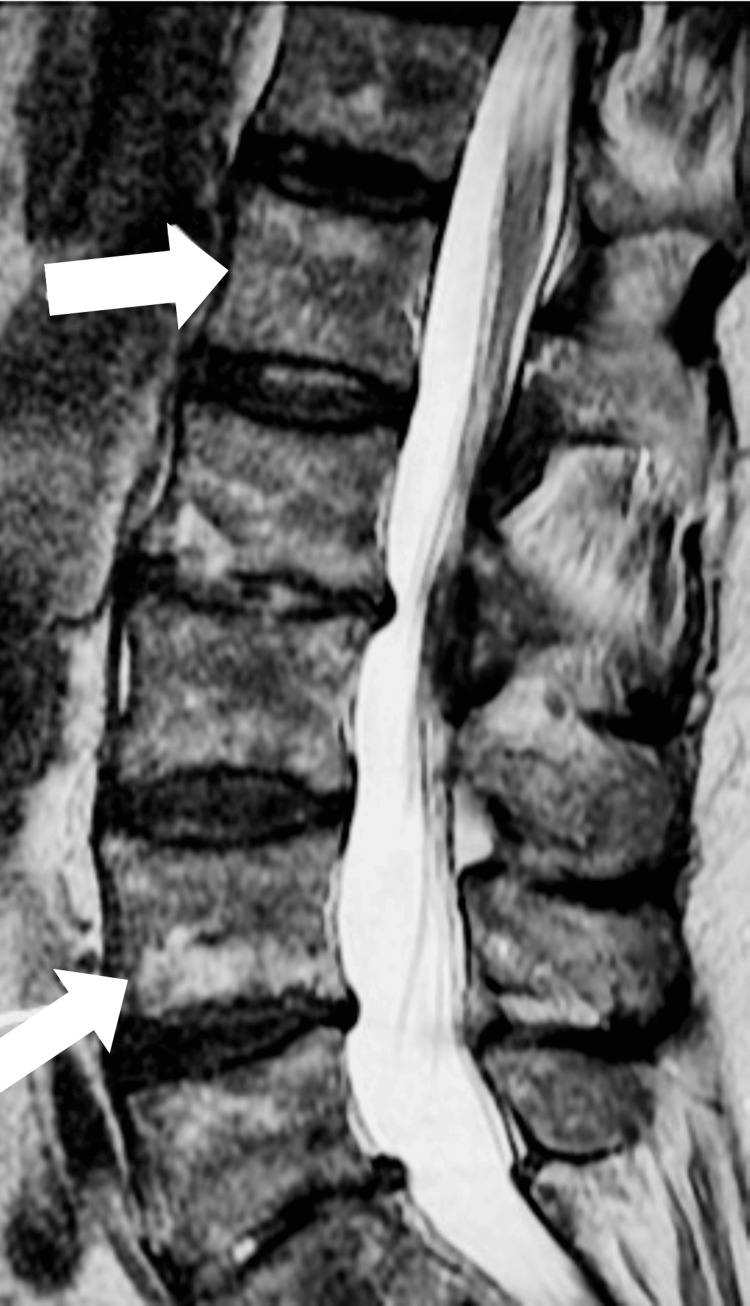
Abnormal T2 sagittal lumbar MRI demonstrating diffuse bone marrow changes and disk degeneration with disk bulges at L5/S1 and L2/3. Arrows mark the diffuse bone marrow changes MRI: magnetic resonance imaging

The chiropractor treated him thrice using distraction manipulation and electrical stimulation. Pain levels ranged from two to eight over the following week. Although the patient reported some relief with care, the pain gradually returned. He is now seven weeks from the initial presentation. An MRI of the thoracic and lumbar spine was ordered, revealing diffuse areas of abnormal bone signals (Figure 3).

**Figure 4:**
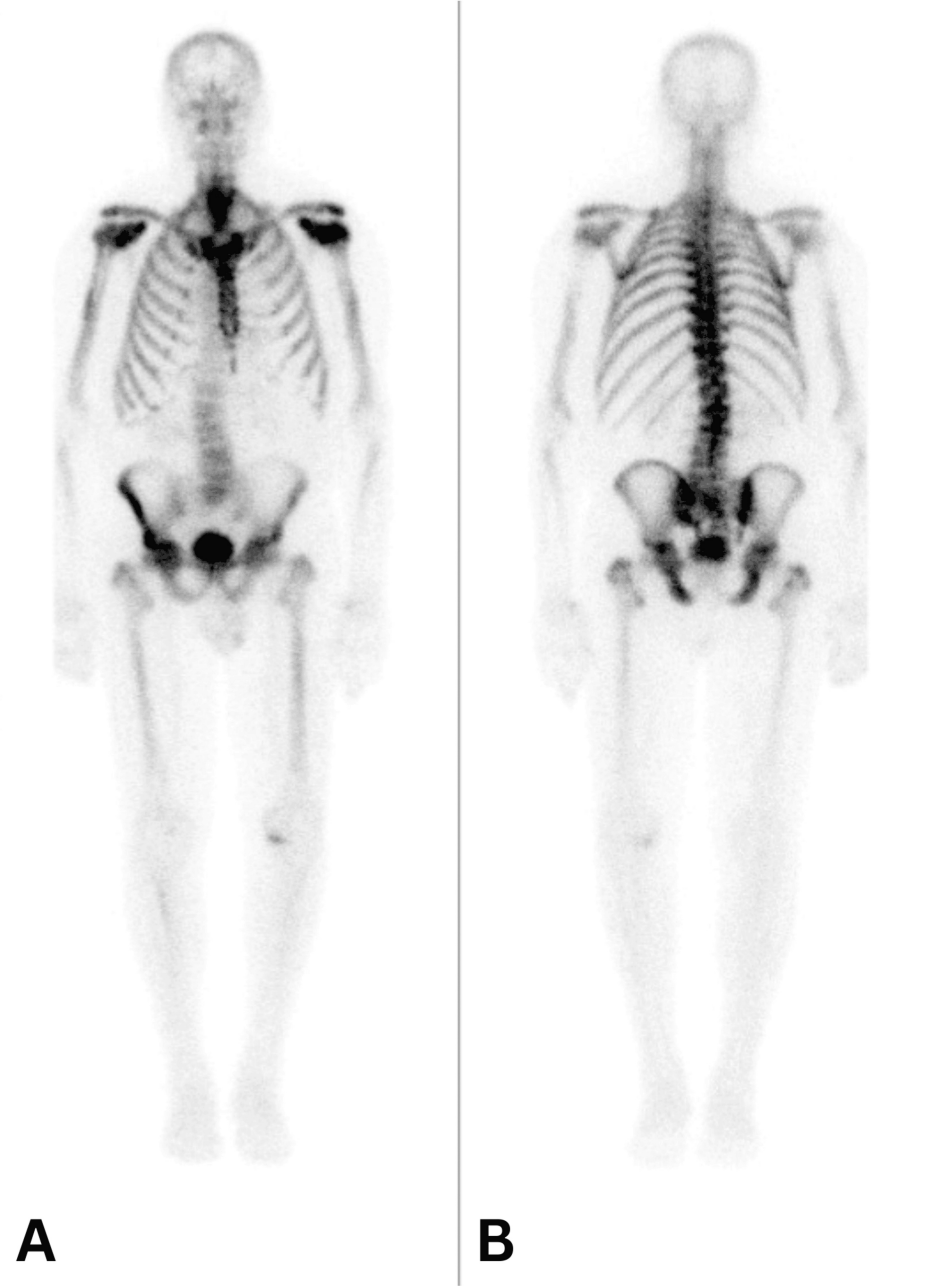
Bone scan demonstrating high bone to soft tissue tracer accumulation: (A) RT anterior LT and (B) LT posterior RT RT: right, LT: left

This was interpreted as being partially due to diffuse degenerative changes, but the differentials included metastasis and multiple myeloma. A 3.8 cm fusiform aneurysm was noted. The spleen appeared larger compared to the previous CT. A bone scan was performed six days later and demonstrated a high ratio of bone to soft tissue radiotracer accumulation. There was no focal abnormal uptake. These findings were consistent with a superscan (Figure 4).

Previous imaging from three years ago was located and showed osteoblastic activity on an MRI scan. The leading diagnosis is strongly considered metabolic bone disease. The patient’s serum parathyroid level was 26.2 pg/ml, whereas the normal range is 15.0-65.0 pg/ml. This minimized the possibility of parathyroid dysfunction as the etiology for the MRI changes.

Based on the above, the chiropractor expressed concerns about multiple myeloma, with metastatic disease being less likely. A consultation with oncology was initiated. The bone biopsy confirmed a diagnosis of myelofibrosis. The time from initial presentation to definitive diagnosis was approximately two months.

As of six months post-diagnosis, our patient has experienced less abdominal pain and continues to live independently. His biggest complaint is fatigue.

## Discussion

Myelofibrosis is a chronic myeloproliferative neoplasm (MPN) that includes chronic myeloid leukemia (BCR-ABL1+), chronic neutrophilic leukemia, chronic eosinophilic leukemia, MPN, primary myelofibrosis (PMF), polycythemia vera, and essential thrombocythemia. Myelofibrosis refers to the fibrosis of the normal spongy bone marrow. It is classified as primary or secondary and is associated with polycythemia vera and thrombocytopenia. PMF is an MPN characterized by stem cell-derived clonal myeloproliferation, which is often, but not always, accompanied by JAK2, CALR, or MPL mutations [6]. Secondary myelofibrosis accounts for about 20% of cases.

As many as 50% of patients have symptoms for up to a year before diagnosis [7]. According to O’Sullivan and Harrison, 30% of patients are asymptomatic [8]. Over 50% of patients have no constitutional symptoms [9]. The most common findings are splenomegaly and anemia in about 90% of patients [9]. This patient exhibited both, and early recognition could have helped expedite the diagnosis. The most common presenting symptom is fatigue [10,11]. Other common complaints include tiredness, abdominal discomfort, shortness of breath, weight loss, and abnormal sweating (Table 2) [9,10].

**Table 2:** Signs and laboratory values recorded in patient notes at diagnosis (n = 200) Hb: hemoglobin [9]

Signs and laboratory values recorded in patient notes at diagnosis	Patients (n = 200)
Splenomegaly	94 (47%)
Anemia	88 (44%)
Leukocytosis	7 (3.5%)
Thrombocytopenia	5 (2.5%)
Thrombocytosis	4 (2%)
Unexplained fever	2 (1%)
Laboratory values^ a^	
Anemia (Hb)	63 (33.0%)
Thrombocytopenia (platelet count <150 × 10^9^/L)	35 (18.3%)
Symptoms recorded in patient notes at diagnosis	
Unexplained tiredness	54 (27%)
Unintended weight loss	42 (21%)
Excessive sweating (especially at night)	31 (16%)
Shortness of breath	18 (9%)
Bone and joint pain	16 (8%)
Feeling of discomfort or abdominal pain	14 (7%)
Fullness, indigestion, and a loss of appetite	14 (7%)
Pruritus	12 (6%)
Weakness	11 (6%)
Bleeding problems	6 (3%)
Palpitations	1 (1%)
Abdominal discomfort from an enlarged liver	1 (1%)
Number of constitutional symptoms recorded in patient notes at diagnosis	
0	114 (57%)
1	45 (23%)
2	15 (8%)
None recorded^ b^	26 (13%)

Myelofibrosis is rare, with about 1.5 cases per 100,000 [10]. In a study of 174 patients with myelofibrosis, the average age of diagnosis was 59.6, with a range of 28-89. Males represented 51% of the cases, and 32% were diagnosed within six months of presentation, while 22% took over two years [11]. Age was not likely a clue in the diagnosis of our patient, as skeletal pain in 76-year-olds is common. Failure to respond to care was a larger clue that something else was the etiology of the pain.

Bone marrow morphology is the primary basis for diagnosis. A JAK2, CALR, or MPL mutation is expected in around 90% of patients [12]. Bone marrow biopsy is the standard diagnostic method but has some limitations, including bone hardness, non-homogeneous fibrosis, and errors in the grading system [13].

A complete blood count panel with careful examination of the peripheral smear is essential in patients with PMF, as peripheral blood can reveal leukoerythroblastosis with teardrop poikilocytosis. A skeletal X-ray shows increased density and a trabecular pattern, which can be mottled. This is highly nonspecific and can include malignancy, myelofibrosis, mastocytosis, sickle cell disease, Paget’s disease of bone, renal osteodystrophy, osteopetrosis, sclerotic dysplasia, osteopoikilosis, osteopathia striata, melorheostosis, hyperthyroidism, hypoparathyroidism, and fluorosis. Diffuse bony sclerosis has multiple causes. Our patient had normal calcium and parathyroid levels.

The average age of diagnosis is around 60-68 [9,11]. Our patient was 76. Myelofibrosis can be diagnosed in children but is estimated to be about 100 times more common in adults [14,15]. About 12% of cases progress to acute myeloid leukemia, an aggressive form of blood cancer. It is characterized by stem cell-derived clonal proliferation that is usually, but not always, associated with mutations [16]. These mutations are classified into JAK2 (60-65%), CALR (20-25%), or MPL (5-10%) mutations, subclonal mutations, and fibrosis on bone marrow biopsies. Typically, early myelofibrosis is characterized by myeloid hypercellularity and abundant atypical megakaryocytes, while fibrosis and osteosclerosis predominate in later stages. Abnormal immature megakaryocytes are the hallmark feature, exhibiting reduced GATA1 protein expression. The pro-inflammatory cytokines secreted include IL-1β, TGF-β, and growth factors bFGF, PDGF, and VEGF, in addition to extracellular matrix components such as fibronectin, laminin, and collagens [17]. The cause of death in 20% of patients is leukemic progression, but comorbidities include cardiovascular events, infection, or bleeding [12]. Bone biopsy is the primary basis for diagnosis, and the presence of JAK2, CALR, or MPL mutations aids the diagnosis but is not essential.

Several different adverse mutations predict inferior survival rates, independent of risk factors. Various adverse karyotypes carry very high risks.

PMF has two prognostic systems: a genetically inspired prognostic scoring system (GIPPS) and a mutation-enhanced international prognostic scoring system 70+ v2.0 (MIPSS70+ v2.0). GIPPS is based on mutations and karyotypes, while MIPSS70+ v2.0 includes clinical risk factors. GIPPS has four risk factors, and MIPSS70+ v2.0 has five.

Survival rates depend on classification. Outcomes vary from observation with a good prognosis to rapidly progressive disease and death. In the observation group, the low-risk disease group has an estimated 10-year survival rate of 56-92%. The survival rate is 30% in the intermediate group and 0-13% in the high-risk group [5]. Our patient was classified in the intermediate group. A stem cell transplant is the only known cure. Treatment for intermediate-risk disease includes JAK2 inhibitors like ruxolitinib and fedratinib, with a survival rate of 30%. Our patient was started on a JAK2 inhibitor and later switched to momelotinib, a recently approved JAK1 and JAK2 inhibitor for intermediate and advanced myelofibrosis. A stem cell transplant is the only option that may prolong survival or be considered a cure [18].

Splenectomy for splenic abdominal pain, symptomatic portal hypertension, thrombocytopenia, and frequent red blood cell transfusions in 314 splenectomized patients showed that 75% reported relief for one year [19]. Our patient did have spleen enlargement but did not undergo a splenectomy. In a follow-up approximately six months after the diagnosis, he reported that his spleen had shrunk by about 50%, and his abdominal pain had decreased.

MRI scans can help differentiate essential thrombocythemia from myelofibrosis. In most cases, the MRI signal in the vertebra was not altered in essential thrombocythemia, whereas it was markedly reduced in myelofibrosis. The difference is that the bone marrow adipose tissue in essential thrombocythemia is grossly preserved, while in myelofibrosis, it is usually markedly decreased or absent [20]. Abnormal vertebral, pelvic, and femoral signal intensities (i.e., hypointense or isointense) on T1-weighted images are the most likely MRI findings, with the vertebra being the most common site.

In our patient, thoracic and lumbar MRIs demonstrated diffuse areas of abnormal bone signal. This was interpreted as partially due to diffuse degenerative changes but included the differentials of metastasis and multiple myeloma.

## Conclusions

Presented is an unusual cause of back pain in a 76-year-old patient. This case emphasizes the uncommon etiology of his pain, which was caused by myelofibrosis. It follows his lack of progress with treatment and the tests performed to uncover the cause of his symptoms. It includes a brief overview of diagnostic clues and treatment options. The case serves as a reminder to healthcare professionals of the importance of maintaining a broad differential diagnosis when evaluating back pain, especially when a patient does not respond to treatment and presents with recurring symptoms.
